# Tax-mediated re-routing of the HTLV-1 p13 protein to nuclear speckles

**DOI:** 10.1186/1742-4690-8-S1-A125

**Published:** 2011-06-06

**Authors:** Vibeke Andresen, Cynthia A Pise-Masison, Uma Sinha-Datta, Robyn Washington Parks, Valentina Cecchinato, Risaku Fukumoto, Christophe Nicot, Genoveffa Franchini

**Affiliations:** 1 Animal Models and Retroviral Vaccines Section, National Cancer Institute, Bethesda, Maryland, 20892, USA; 2 Department of Pathology and Laboratory Medicine, University of Kansas Medical Center, Kansas City, Kansas, 66160, USA

## 

Human T-cell Leukemia Virus type-1 (HTLV-1) is highly immunogenic and has low variability so tight regulation of its expression is essential for virus maintenance in vivo. HTLV-1 replication is positively regulated by Tax and Rex, and negatively by the p30 and HBZ proteins. Here, we demonstrate that HTLV-1 encodes another negative regulator of virus expression, the p13 protein. Expressed separately, p13 localizes to the mitochondria, but in the presence of Tax, p13 is stabilized, and re-routed to the nuclear speckles. The p13 protein directly binds Tax, decreases Tax binding to the CBP/p300 transcriptional co-activator and, by reducing Tax transcriptional activity, suppresses viral expression. These findings suggest that HTLV-1 has evolved a complex mechanism to control its own replication through regulation of positive and negative viral proteins. Further, these results emphasize the importance of studying the function of the HTLV-1 viral proteins, not only in isolation, but also in the context of full viral replication.

